# The Anti-Proliferative Effect of PI3K/mTOR and ERK Inhibition in Monolayer and Three-Dimensional Ovarian Cancer Cell Models

**DOI:** 10.3390/cancers14020395

**Published:** 2022-01-13

**Authors:** Elizabeth Dunn, Kenny Chitcholtan, Peter Sykes, Ashley Garrill

**Affiliations:** 1 School of Biological Sciences, University of Canterbury, Christchurch 8041, New Zealand; 2 Department of Obstetrics and Gynaecology, University of Otago, Christchurch 8011, New Zealand; kenny.chitcholtan@otago.ac.nz (K.C.); peter.sykes@otago.ac.nz (P.S.); 3 Biomolecular Interaction Centre, School of Biological Sciences, University of Canterbury, Christchurch 8041, New Zealand

**Keywords:** ovarian cancer, combination chemotherapy, three-dimensional model, spheroids, BEZ235, dactolisib, SCH772984

## Abstract

**Simple Summary:**

In ovarian cancer patients the PI3K/AKT/mTOR and RAS/RAF/MEK/ERK kinase signaling pathways are frequently dysregulated, making them potential targets of therapeutic inhibitors. In this study, we used four human ovarian cancer cell lines grown in two- and three-dimensional models to investigate the potential efficacy of combining two inhibitors, which target these pathways, against ovarian cancer. The inhibitor combination was found to have cell line- and model-dependent synergistic antiproliferative effect.

**Abstract:**

Most ovarian cancer patients are diagnosed with advanced stage disease, which becomes unresponsive to chemotherapeutic treatments. The PI3K/AKT/mTOR and the RAS/RAF/MEK/ERK kinase signaling pathways are attractive targets for potential therapeutic inhibitors, due to the high frequency of mutations to PTEN, PIK3CA, KRAS and BRAF in several ovarian cancer subtypes. However, monotherapies targeting one of these pathways have shown modest effects in clinical trials. This limited efficacy of the agents could be due to upregulation and increased signaling via the adjacent alternative pathway. In this study, the efficacy of combined PI3K/mTOR (BEZ235) and ERK inhibition (SCH772984) was investigated in four human ovarian cancer cell lines, grown as monolayer and three-dimensional cell aggregates. The inhibitor combination reduced cellular proliferation in a synergistic manner in OV-90 and OVCAR8 monolayers and in OV-90, OVCAR5 and SKOV3 aggregates. Sensitivity to the inhibitors was reduced in three-dimensional cell aggregates in comparison to monolayers. OV-90 cells cultured in large spheroids were sensitive to the inhibitors and displayed a robust synergistic antiproliferative response to the inhibitor combination. In contrast, OVCAR8 spheroids were resistant to the inhibitors. These findings suggest that combined PI3K/mTOR and ERK inhibition could be a useful strategy for overcoming treatment resistance in ovarian cancer and warrants further preclinical investigation. Additionally, in some cell lines the use of different three-dimensional models can influence cell line sensitivity to PI3K/mTOR and RAS/RAF/MEK/ERK pathway inhibitors.

## 1. Introduction

Ovarian cancer is a deadly disease that has a five-year survival rate below 50% [1]. Although ovarian cancer mortality has decreased due to a reduction in incidence (10 deaths per 100,000 in 1976 to 6.7 per 100,000 in 2015), the five-year survival rates have changed little in the past few decades [1]. This is due in part to 70% of ovarian cancer patients initially being diagnosed with an advanced stage of the cancer, which is a result of a lack of effective screening methods and of initial symptoms that are often attributed to other less critical conditions [1,2]. Additionally, ovarian cancer is a highly heterogeneous disease with different subtypes that have distinct genotypic and phenotypic characteristics [1,3]. Therefore, successful treatment of ovarian cancer is likely to require an arsenal of different treatment options, which can be tailored to each patient with specific biomarkers.

Mutations within the PI3K/AKT/mTOR and RAS/RAF/MEK/ERK kinase signaling pathways are characteristic of the low-grade serous (LGSOC), mucinous (MOC), endometrioid (ENOC) and clear cell (CCOC) ovarian cancer subtypes [4,5,6,7]. Both of these pathways play critical roles in normal healthy cells and their dysregulation can result in excessive proliferation and cell survival [8,9,10]. In addition, upregulation of these pathways has been implicated in the evasion of anoikis, the loss of adhesion and the promotion of invasion, thus enabling intraperitoneal metastasis [11,12,13,14].

The PI3K/AKT/mTOR signaling pathway is thought to be aberrant in approximately 70% of ovarian cancers [15]. Inactivating mutations to phosphatase and tensin homologue deleted on chromosome ten (PTEN) occur in 3–8% [15], loss of heterozygosity occurs in 27% [16] and mutations to the phosphatidylinositol-3-kinase 110-kDa catalytic subunit *PIK3CA* gene occurs in 6–12% [17,18] of cases. Furthermore, increased activity of the pathway has been associated with resistance to both chemotherapy [19,20] and radiation therapy [21]. Several studies have investigated inhibitors of the pathway in both in vitro and in vivo ovarian cancer cell models and in clinical trials [22,23,24,25]. Pan class phosphoinositide 3-kinase (PI3K) inhibitors have shown anti-proliferative effects in both wild-type and mutant *PIK3CA* ovarian cancer cells in vitro [22,23]. Additionally, these inhibitors, when used in combination with traditional chemotherapy agents, poly(ADP-ribose) polymerase (PARP) inhibitors and other tyrosine kinase inhibitors, have been shown to reduce cell proliferation and induce apoptosis in a synergistic manner [19,22,25,26]. Unfortunately, PI3K and mammalian or mechanistic target of rapamycin (mTOR) inhibitors have been shown to increase the expression of active phosphorylated extracellular signal-related kinase (p-ERK) and phosphorylated protein kinase B (p-AKT) in in vitro and in vivo ovarian cancer cell models, indicating that cancer cells have or develop resistance mechanisms to these inhibitors, likely through the loss of negative feedback loops [22,27,28,29].

BEZ235 is a dual PI3K and mTOR inhibitor that has shown considerable efficacy against a wide range of different cancer cell lines including ovarian, breast and bladder cancers [29,30,31]. In combination treatments BEZ235 has been shown to overcome resistance to epidermal growth factor receptor (ErbB) inhibitors in breast cancer [32], to sensitize ovarian carcinoma cells to anti-apoptosis inhibitors [33], to enhance sensitivity to PARP inhibitors in breast cancer cells and to re-sensitize cells to cisplatin in bladder and ovarian cancer cell lines [29,31]. Unfortunately, clinical trials with BEZ235/Dactolisib have had toxicity issues, which have prevented the assessment of tumor response [34,35]. Despite the observed toxicity effects, which compromises the sole use of this inhibitor, it could potentially be used in the clinic in combination treatments, where, if synergism occurs, the dose and thus toxicity, may be reduced without compromising efficacy.

Increased activation of the RAS/RAF/MEK/ERK signaling pathway occurs in approximately 40% of ovarian cancers, most commonly in low-grade ovarian cancers [7,36]. Mutations to Kirsten rat sarcoma virus (KRAS) and serine/threonine-protein kinase B-Raf (BRAF) are found in approximately 14% and 9% of cases, respectively, [36]. Mitogen-extracellular activated protein kinase (MEK) inhibitors have shown efficacy in in vitro [37] and in vivo models [36] as well as in clinical trials [38]. MEK inhibitors have also been investigated in combination with inhibitors that increase the expression of BH3 pro-apoptosis proteins and inhibit anti-apoptosis proteins (Bcl-2, Bcl-xL) resulting in a greater reduction in proliferation and survival in ovarian cancer cell lines [39]. Unfortunately, resistance to MEK inhibitors has been reported in both preclinical and clinical studies [37,40].

SCH772984, which was developed to treat BRAF and MEK inhibitor resistant melanoma, is a highly specific inhibitor of ERK [41]. It has been shown to induce apoptosis and cell cycle arrest and decrease p-ERK expression in melanoma cell lines including cell lines that are resistant to BRAF inhibitors [42] and ERK inhibition has been found to be more effective than MEK inhibition at overcoming resistance to BRAF inhibitors in melanoma cells [43]. Melanoma cell lines have shown increased p-AKT levels in response to SCH772984 treatment [42], suggesting that combining this inhibitor with a PI3K/AKT pathway inhibitor may be an effective strategy. Although ERK inhibitors have not been investigated in ovarian cancer cell lines, MEK inhibitors have shown efficacy in several ovarian cancer monolayer models [36]. KRAS and BRAF mutations are a predictor of sensitivity to MEK inhibitors in ovarian cancer cell lines [25].

The clinical relevance of the responsiveness of ovarian cancer cells to anti-cancer drugs may be influenced by the unique features and microenvironment niche of those cells [44]. In the advanced stage of disease single cells, small clusters, large aggregates and spheroids are found in the peritoneal cavity, which is filled with ascitic fluid containing growth factors, cytokines and other factors that influence the progression and treatment sensitivity of the disease [45]. The mechanisms driving the survival of these unique cell populations is poorly defined. Therefore, it is important to replicate and use these cell clusters, aggregates and spheroids in in vitro models to uncover the mechanisms of action of drug targets as well as potential sources of resistance to these drugs. Cell clusters, aggregates and spheroids are classified as three dimensional (3D) models and are considered superior to monolayer models as they are thought to better recapitulate the microenvironment and drug sensitivity of actual tumors [46]. Three-dimensional (3D) models can have cell–cell and cell–extracellular matrix (ECM) interactions, nutrient, waste and drug penetration layers, necrotic and quiescent regions, decreased proliferation, restored histological features, and differential responses to therapeutics [46,47,48].

Complexity can be added to in vitro cancer models to provide a better representation of tumors and their response to treatments. Examples of these include simple forced suspension and hanging drop models, which restore the microenvironment features outlined above, and more complex co-culture systems, with the inclusion of stromal cells, which influence the development and progression of cancers, and ECM constituents, which can impact gene expression, cell growth and survival and reduce the penetration of anticancer drugs into tumors [49,50]. These models can however have disadvantages, including increased cost, time and may require specialized equipment as well as presenting challenges associated with extracting, imaging and assaying the cancer cells and they may also reduce reproducibility [51]. New assays and technologies are being developed to enhance the monitoring of cells in three-dimensional models, which will mitigate some of these issues [52]. The relevance of ECM-mimicking hydrogels can also be challenged given the diverse and dynamic nature of the ECM in tumors, while natural hydrogels, such as matrigel, have issues with batch-variation and reproducibility [51]. Three-dimensional cancer models enable the investigation of the effect of the microenvironment, including ECM stiffness and stromal cell interactions, on cancer progression [50,53,54]. The synergistic effects of drug combinations have been shown to be dependent on whether a monolayer or spheroidal model was used [55].

The anti-tumor effect of kinase inhibitors targeted at the PI3K/AKT/mTOR and RAS/RAF/MEK/ERK pathways is likely to be anti-proliferative rather than through the promotion of cell death (cytostatic vs. cytotoxic). This means that the research outcomes may be much more promising in 2D monolayers, where proliferation is more pronounced, than in 3D models. Additionally, the gene expression profiles of these pathways are different in 3D models and are thought to be more representative of in vivo models than 2D monolayer models [47]. While, it has been well established that testing of traditional cytotoxic chemotherapeutic in 3D cancer models reduces their apparent efficacy in comparison to 2D monolayer models, the effect of culture model on the efficacy of treatments targeting proliferation and survival signaling pathways has yet to be well established in ovarian cancer models. This is likely to have important implications for the determination of effective biomarkers, which will be critical to stratifying ovarian cancer patients to match the appropriate treatment for their disease.

This study aims to provide a comparison of the antiproliferative effect of PI3K/AKT/mTOR and RAS/RAF/MEK/ERK pathway inhibitors in 2D monolayer and 3D ovarian cancer models. The PI3K/mTOR inhibitor BEZ235/Dactolisib and the ERK inhibitor SCH772984 were used, both alone and in combination. Additionally, the impact of culture model on findings of synergistic interactions of these inhibitors was investigated. We hypothesise that the synergistic effects of the PI3K and ERK inhibitors on ovarian cancer cells is cell line- and cell model-dependent.

## 2. Materials and Methods

### 2.1. Cell Lines, Media and Culturing

The four human ovarian adenocarcinoma cell lines, OV-90, OVCAR5, OVCAR8, and SKOV3 were kindly donated by Dr Kenny Chitcholtan (Department of Obstetrics and Gynaecology, University of Otago, Christchurch, New Zealand) and had been authenticated using STR testing by CellBank (Children’s Medical Research Institute, New South Wales, Australia). These were maintained in Dulbecco’s Modified Eagle Medium nutrient mix F12 (DMEM-F12) (Catalogue number 10091148, GIBCO${}^{TM}$, Life Technologies, Auckland, New Zealand) supplemented with 5% (*v*/*v*) foetal bovine serum (FBS) (GIBCO${}^{TM}$, Life Technologies, Auckland, New Zealand), 1% PenStrep (50 units/mL penicillin and 50 μM streptomycin), 2 mM GlutaMAX and 2 μg/mL amphotericin B (all GIBCO${}^{TM}$, Life Technologies, Auckland, New Zealand), which is henceforth referred to as working media. OV-90 working media was additionally supplemented with 5% (*v*/*v*) Knockout${}^{TM}$ Serum Replacement (GIBCO${}^{TM}$ Life Technologies, New Zealand). Cultures were maintained in 25 cm${}^{2}$ and 75 cm${}^{2}$ cell culture flasks (CELLSTAR TC, Lab Supply, Dunedin, New Zealand) in a humidified 5% CO${}_{2}$ incubator.

Media was replaced as required (every 2–3 days) and cells were sub-cultured when near confluent. Cells were subcultured by discarding the working media and rinsing with phosphate-buffer solution (PBS) before incubation with 1× trpsin- ethylenediaminetetraacetic acid (trypsin-EDTA) (Life Technologies, New Zealand) until the cells became detached (after approximately 10 min). Cells suspended in trypsin-EDTA were collected in 15 or 50 mL tubes, flasks were rinsed with PBS and that PBS was added to the tubes before centrifugation at 0.2 rcf for 5 min. The supernatant was then discarded, and the cell pellet was resuspended in working media and a portion (typically 1/8th) was added to culture flasks and/or counted and added to well plates for subsequent experiments.

### 2.2. Generation of 2D Monolayer and 3D Aggregate Cultures

Experiments involving the generation of 2D monolayer and 3D aggregate cultures were carried out in 24 well tissue culture plates (BIOFIL, Lab Supply, New Zealand). Cells were collected when near confluent by trypsinisation as outlined above. The approximate number of viable cells in the cell suspension was determined on a haemocytometer using trypan blue exclusion (Gibco${}^{\mathrm{TM}}$, Life Technologies, New Zealand) observed using a Nikon Eclipse Ts2 microscope. Two-dimensional (2D) cultures plates were seeded with 5 × 10${}^{4}$ cells per well. 3D cell culture was carried out on poly-hydroxyethylmethacrylate (poly-HEMA) (Sigma-Aldrich, New Zealand) coated 24 well plates. Poly-HEMA was dissolved in 95% ethanol at a concentration of 12 mg/mL by heating to 70 ${}^{\circ }$C for approximately one hour to ensure all of the polyHEMA was dissolved. 250 μL of polyHEMA solution was added to each well and the plates were left overnight on a 50 rpm shaker table at 37 ${}^{\circ }$C until the ethanol evaporated. Prior to cell seeding, the polyHEMA coated well plates were rinsed with 250 μL of sterile PBS for at least 5 min. Cells were seeded at 1 × 10${}^{5}$ cells per well for 3D cell aggregates. The working media volume in each well was adjusted to 1 mL for both monolayer and 3D aggregate cultures. Cells were established in the 24 well plates for two or seven days at 37 ${}^{\circ }$C in a 5% CO${}_{2}$ incubator prior to treatment initiation.

### 2.3. Treatment with BEZ235 and SCH772984

Forty eight hours after establishment, monolayers and 3D cell aggregates were treated with BEZ235 and SCH772984 (ApexBio, Houston, TX, USA), alone or in combination, for 72 h, or for 24 h for the apoptosis assays. To make up stock inhibitor solutions, BEZ235 and SCH772984 were dissolved in dimethylsulfoxide (DMSO) (Sigma-Aldrich, New Zealand) to a concentration of 10 mM. In each experiment all treatments had the same DMSO concentration which was equal to the vehicle control. In general, the DMSO concentration was kept at or below 0.1% (v/v) except in the combination experiments for SKOV3 monolayers (0.2%) and OVCAR8 (0.56%), OVCAR5 (0.52%) and SKOV3 (0.47%) aggregates (due to their high EC${}_{50}$ values) and in the single inhibitor treatments of aggregates and spheroids where the concentration of DMSO was at 0.5% (*v*/*v*) (due to a higher concentration of 50 µM treatment being used). The stock solutions were diluted with working media to a 2× treatment concentration. Five hundred μL (i.e., one half the volume) of the working media was removed from each well to ensure that 3D aggregate cultures were not perturbed during the addition of the inhibitor. Five hundred μL of the 2× treatment was then added, to give a final concentration of 1×. Concentrations of 0.05, 0.1, 0.5, 1, 5 and 10 μM were used in proliferation experiments and half-maximal effective concentration (EC${}_{50}$) values were predicted using Compusyn software [56].

### 2.4. Trypan Blue Exclusion Assays to Measure Proliferation

Following 72 h of treatment the numbers of viable cells in each treatment group was determined by counting the cells on a haemocytometer using the Trypan Blue exclusion assay observed through a BH2 Olympus microscope. For the 2D monolayers, cells were rinsed with PBS then detached from the well plates by trypsinization at 37 ${}^{\circ }$C. Detached cells were then transferred into 1.5 mL microtubes and rinsed with PBS by pelleting the cells and discarding the supernatant. Cells were then resuspended in 50 μL of PBS and stored on ice.

Three-dimensional (3D) cell aggregates were collected in 1.5 mL microtubes and the media discarded by pelleting the aggregates and discarding the supernatant and rinsing once with PBS. Aggregates were then trypsinized at 37 ${}^{\circ }$C to break apart aggregates, after which, they were rinsed twice with PBS before resuspension in 50 μL of PBS and then stored on ice.

Prior to counting of the cells, 50 μL of 0.4% Trypan Blue was added to the 50 μL cell suspension and the cell suspension was loaded on to the haemocytometer. The percentage of viable cells relative to the control was used as an indication of proliferation in each treatment. All experiments contained three replicates for each treatment. Each experiment was repeated a minimum of three times.

### 2.5. Determination of Synergistic Interactions

Synergistic interactions between BEZ235 and SCH772984 were determined using the methodologies described by Chou [56]. CompuSyn software was used to distinguish synergistic, additive or antagonistic effects from the combination BEZ235 and SCH772984. This gives a quantitative means to determine drug interactions through the combination index (CI) value, where CI = 1 indicates additive effects, CI > 1 antagonistic effects and CI <1 indicates synergism. Briefly, in most instances BEZ235 and SCH772984 were combined as a stock solution that was 4× (EC${}_{50}$)${}_{\mathrm{BEZ}235}$:(EC${}_{50}$)${}_{\mathrm{SCH}772984}$ and then serially diluted 2-fold to give treatment concentrations of 1/4, 1/2, 1, 2 and 4× (EC${}_{50}$)${}_{\mathrm{BEZ}235}$:(EC${}_{50}$)${}_{\mathrm{SCH}772984}$. Using this approach each cell line was treated with a different combination and that combination also differed between monolayer and cell aggregates. In instances (OVCAR5 and SKOV3 3D aggregates) where this combination strategy would have led to excessive DMSO a 1× EC${}_{50}$ stock solution was made and serially diluted to give treatment concentrations of 1/16, 1/8, 1/4, 1/2 and 1× (EC${}_{50}$)${}_{\mathrm{BEZ}235}$:(EC${}_{50}$)${}_{\mathrm{SCH}772984}$. Additionally, for SKOV3 3D aggregate the EC${}_{50}$ value for SCH772984 was unobtainable due to solubility limits and, therefore, the OVCAR5 3D aggregate value was used as this was the next highest value. Henceforth, the combinations will be abbreviated to 1/2, 1× etc EC${}_{50}$ combination. In experiments comparing the effect of the inhibitor combinations on OV-90 aggregates and spheroids, the predicted EC${}_{50}$ values from the spheroids were used to determine the concentrations for treating both the aggregates and spheroids. Therefore, both aggregates and spheroids were treated with the same concentration of inhibitors enabling a more direct comparison between the two models.

### 2.6. Hanging Drop Culture for Spheroids

A simple hanging drop technique was adapted from that of Foty [57]. Following trypsinization, the cell suspension was collected, and media was added to halt trypsinization, cells were then pelleted and the trypsin was discarded. The cells were rinsed with their culture media and re-pelleted. The media was then discarded, and the cells were resuspended in fresh media. Cell density was determined on a haemocytometer and the suspension was diluted to give a cell concentration of 1.25 × 10${}^{6}$ cells/mL. Twenty μL drops of cell suspension were added to the lid of 24 well plates above the 8 internal wells, so that each drop had approximately 2.5 × 10${}^{4}$ cells. Wells were filled with 1 mL of PBS to minimise evaporation. Six hanging drops were generated for each treatment. Media was replenished after 4 days. After 7 days the hanging drops media was changed by removing and replenishing 10 μL of media twice so that the spheroids were always suspended in media and were minimally disturbed. Ten μL of media was then removed leaving the drops in 10 μL and then 10 μL of a 2× concentration of inhibitors was added to form a hanging drop of 20 μL at the correct concentration.

### 2.7. Determination of Changes in Size and Morphology of the Hanging Drop Aggregates

Images of individual hanging drop spheroids were captured using a Nikon Eclipse Ts2 inverted microscope (4×/0.10 NA objective lens). The equivalent diameter and sphericity of each spheroid was then determined using AnaSP computer software (Piccinini [58]). To determine the effect of BEZ235 and SCH772984, alone and in combination, on the size and morphology of OV-90 spheroids images were taken prior to treatment (6 spheroids per treatment and 3 biological replicates) and after 72 h of treatment. The AnaSP computer software was used to determine the equivalent diameter (diameter of a circle with the same area as the spheroid section) and sphericity (similarity to a sphere) and the changes over time were determined as described by Zanoni et al. [59]. Following image collection, the spheroids were trypsinized and the number of viable cells were counted using trypan blue exclusion as described above.

### 2.8. Propidium Iodide Staining and Sectioning

To confirm and compare the 3D morphology of OV-90 and OVCAR8 aggregates, the aggregates were first established on polyHEMA-coated plates for 7 days. Aggregates were then stained for one hour at 37 ${}^{\circ }$C 5% CO${}_{2}$ with propidium iodide (PI) (5 μg/mL) and Hoeschst 33342 (10 μM) before fluorescent imaging using a Leica SP5 confocal microscope.

For sectioning the hanging drop spheroids and polyHEMA aggregates were rinsed with PBS and stained for one hour with PI (5 μg/mL). Spheroids were then rinsed five times, fixed for 20 min in 50% methanol and 50% acetone, stained with 0.5% aniline blue for 20 min, rinsed with PBS and embedded in optimal cutting temperature compound (OCT) before freezing overnight at −80 ${}^{\circ }$C. Eight μm sections were taken every 50 μm using a Leica CM1860 cryostat (Leica Biosystems, Wetzlar, Germany). The middle sections were stained with Hoechst 33342 (2 μm) for 20 min in the dark at 37 ${}^{\circ }$C. Images were collected using a Zeiss Axioimager Z1 microscope (AxioVision 4.5. Apotome software, Carl Zeiss, Oberkochen, Germany) using DAPI and Texas Red fluorescent channels.

### 2.9. Annexin-V FITC and Propidium Iodide Staining for Apoptosis

Cell monolayers and cell aggregates were established for 48 h before treatment with the DMSO control, BEZ235 or SCH772984 alone at their respective EC${}_{50}$ concentrations or a combination at 1/2× or 1× EC${}_{50}$ concentrations. Following 24 h of treatment, monolayer cells were rinsed with PBS, trypsinized for 20 min at 37 ${}^{\circ }$C and collected in 1.5 mL tubes with PBS. Trypsin was removed by pelleting cells and discarding supernatant. Cells were then rinsed with PBS and then with binding buffer. Cell aggregates were collected in 1.5 mL tubes and the media removed by pelleting cells and discarding supernatent. Aggregates were rinsed with PBS and disassociated with 100 μL of trypsin-EDTA at 37 ${}^{\circ }$C. Cells were rinsed twice with PBS and rinsed once with binding buffer (Abcam, Melbourne, Australia). Both monolayer and aggregate cells were resuspended in fresh 200 μL of binding buffer. Cells were counted on a haemocytometer to confirm 1–5 × 10${}^{5}$ cells and diluted if necessary. 2 μL of 500× annexin V-FITC (Abcam, New Zealand) and 2 μL of 250 μg/L propidium iodide were added and incubated in the dark at room temperature for 15 min. The 200 μL of cell suspension was transferred to a 96 well plate then annexin V-FITC and propidium iodide binding was quantified using flow cytometry (Cytomics FC 500 MPL, Beckman Coulter). All experiments contained two technical replicates for each treatment. Each experiment was repeated three times.

### 2.10. Statistical Analysis

Statistical analysis was carried out using GraphPad Prism (GraphPad Software, San Diego, CA, USA). Significance was determined by one-way or two-way ANOVA with Dunnett’s and Tukey’s tests for multiple comparisons. Normality was determined by Shapiro-Wilk test with percentage and ratio data log transformed. The statistical test used to determine significance is indicated in the figure legend. A minimum of three biological replicates were carried out for each experiment.

## 3. Results

### 3.1. The Effects of BEZ235 on Cell Viability in Cell Monolayers and Aggregates of OV-90, OVCAR8, OVCAR5 and SKOV3 Cell Lines

**Figure 1:**
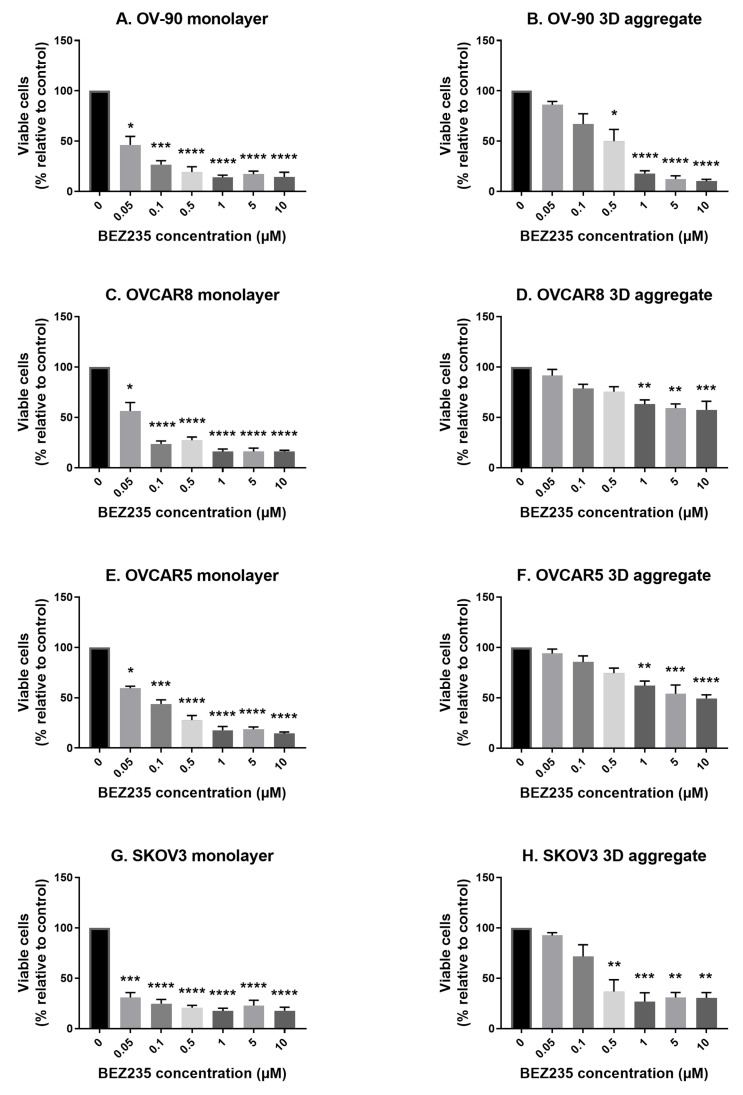
The effect of the dual phosphoinositide 3-kinase (PI3K) and mechanistic target of rapamycin (mTOR) inhibitor BEZ235 on cell viability in OV-90 (**A**,**B**), OVCAR8 (**C**,**D**), OVCAR5 (**E**,**F**) and SKOV3 (**G**,**H**) monolayer and aggregate cultures. Cell viability was significantly reduced, relative to the control, at concentrations of 0.05 (OV90, OVCAR8, OVCAR5 and SKOV3 monolayers), 0.5 (OV90 and SKOV3 aggregates) and 1 μM (OVCAR8 and OVCAR5 aggregates) and above. Cultures were established for 48 h prior to a 72 h exposure to BEZ235 at the concentrations shown. Cell viability was determined by staining cells with trypan blue and conducting a cell count. Data are expressed as mean ± SEM (n = 4). Statistical significance is at *p* < 0.05 and is in comparison to the control. The statistically significant differences are indicated as *p* < 0.05 (*), *p* < 0.01 (**), *p* < 0.001 (***) and *p* < 0.0001 (****), as determined by one-way ANOVA with a Dunnett’s multiple comparison test on log transformed data.

To investigate the effect of the dual PI3K/mTOR inhibitor BEZ235 on cell viability, OV-90, OVCAR8, OVCAR5 and SKOV3 cells were cultured for 48 h as both monolayers and cell aggregates and then exposed to the inhibitor for 72 h. BEZ235 significantly reduced cell viability relative to the controls in all four cell lines and was more effective against monolayers than aggregates (Figure 1). Significant reductions were observed at concentrations of 0.05 (OV90, OVCAR8, OVCAR5 and SKOV3 monolayers), 0.5 (OV90 and SKOV3 aggregates) and 1 μM (OVCAR8 and OVCAR5 aggregates) and above. The EC${}_{50}$ values for each of the four cell lines cultured as monolayers or aggregates are summarized in (Table 1). Overall, higher predicted EC${}_{50}$ values for 3D aggregates indicate that these have reduced sensitivity to BEZ235 in comparison to monolayer cultured cell lines.

### 3.2. The Effect of SCH772984 on Cell Viability in Monolayers and Aggregates of OV-90, OVCAR8, OVCAR5 and SKOV3 Cell Lines

**Figure 2:**
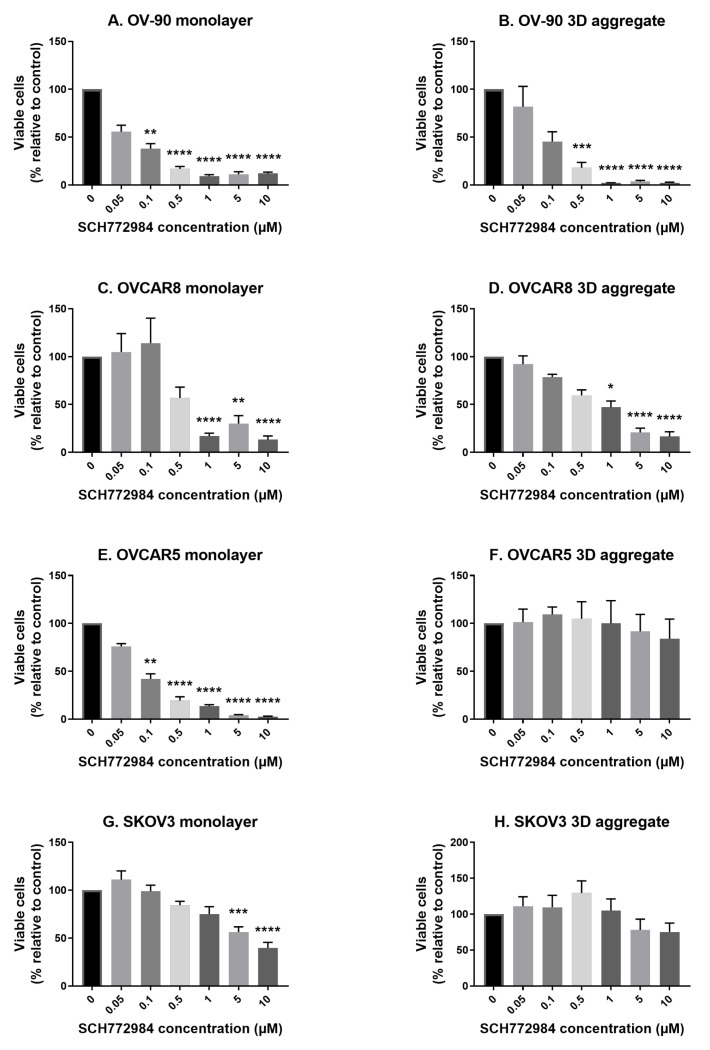
The effect of the extracellular signal-related kinase (ERK) inhibitor SCH772584 on cell viability in OV-90 (**A**,**B**), OVCAR8 (**C**,**D**), OVCAR5 (**E**,**F**) and SKOV3 (**G**,**H**) monolayer and aggregate cultures. Cell viability was significantly reduced, relative to the control, at concentrations of 0.1 μM (OV-90 and OVCAR5 monolayers), 0.5 μM (OV-90 aggregates), 1 μM (OVCAR8 monolayers and aggregates) and 5 μM (SKOV3 monolayers) and above. There was no significant effect on OVCAR5 or SKOV3 aggregates. Cultures were established for 48 h prior to a 72 h exposure to SCH772584 at the concentrations shown. Cell viability was determined by staining cells with trypan blue and conducting a cell count. Data are expressed as mean ± SEM (n = 4). Statistical significance is at *p* < 0.05 and is in comparison to the control. The statistically significant differences are indicated as *p* < 0.05 (*), *p* < 0.01 (**), *p* < 0.001 (***) and *p* < 0.0001 (****), as determined by one-way ANOVA with a Dunnett’s multiple comparison test on log transformed data.

Using the same approach as with BEZ235, monolayer and aggregate cultures of the four cell lines were next exposed to the ERK inhibitor SCH772984. Overall the cell lines were less sensitive to SCH772984, with significant reductions in cell viability relative to the control at concentrations of 0.1 μM (OV-90 and OVCAR5 monolayers), 0.5 μM (OV-90 aggregates), 1 μM (OVCAR8 monolayers and aggregates) and 5 μM (SKOV3 monolayers) and above (Figure 2). The inhibitor had no significant effect on the viability of OVCAR5 or SKOV3 cell aggregates. Again, the EC${}_{50}$ values of all four cell lines in both monolayers and aggregates are summarised in (Table 1).

### 3.3. The Effect of the Combination of BEZ235 and SCH772984 on Cell Viability in Monolayers and Aggregates of OV-90, OVCAR8, OVCAR5 and SKOV3 Cell Lines

**Figure 3:**
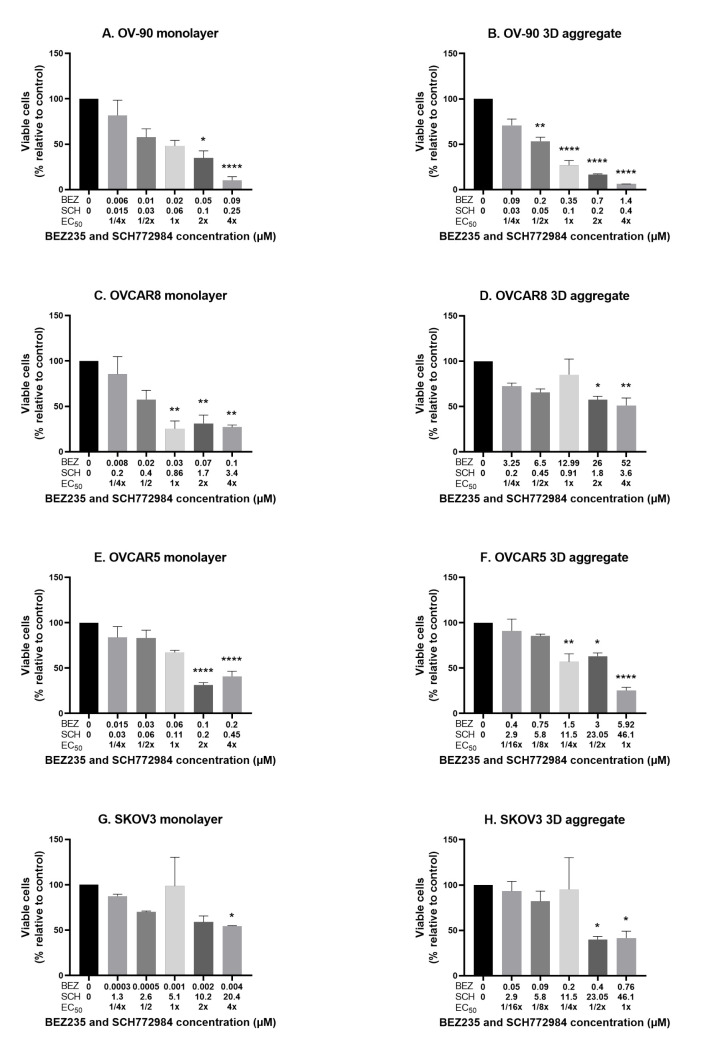

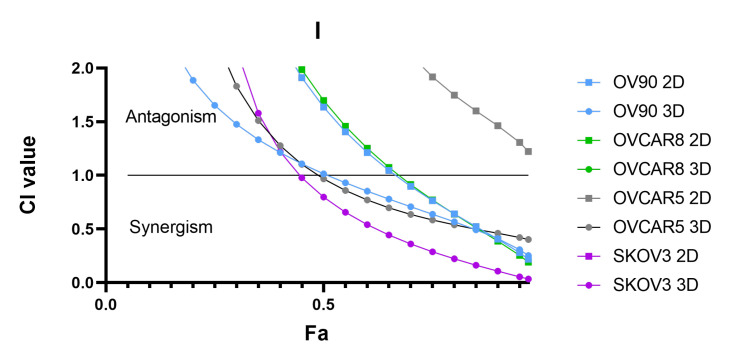
The effect of the combination of BEZ235 and SCH772984 at 1/4, 1/2, 1, 2 and 4× EC${}_{50}$ on cell proliferation in OV-90 (**A**,**B**), OVCAR8 (**C**,**D**), OVCAR5 (**E**,**F**) and SKOV3 (**G**,**H**) monolayer and aggregate cultures. Due to an unobtainable EC${}_{50}$ value (1322.37 μM) for SCH772984 in SKOV3 aggregates the concentration used is based off OVCAR5 aggregates, which also had a high EC${}_{50}$ value (46.1 μM). Concentrations have been rounded for brevity. A significant decrease in proliferation was seen in OV-90, OVCAR8 and OVCAR5 monolayers and in OV-90, OVCAR8, OVCAR5 and SKOV3 aggregates. CI values indicate a synergistic interaction in OV-90 and OVCAR8 monolayers and OV-90, OVCAR5 and SKOV3 3D aggregates (**I**). CI values for SKOV3 monolayers and OVCAR8 3D aggregates do not appear in the figure as these exceeded the range of values used, which indicates a strongly antagonistic effect. Data are expressed as mean ± SEM (n = 4). Statistical significance is at *p* < 0.05 and is in comparison to the control. The statistically significant differences are indicated as *p* < 0.05 (*), *p* < 0.01 (**) and *p* < 0.0001 (****), as determined by one-way ANOVA with Tukey’s multiple comparison test on log transformed data.

**Figure 4:**
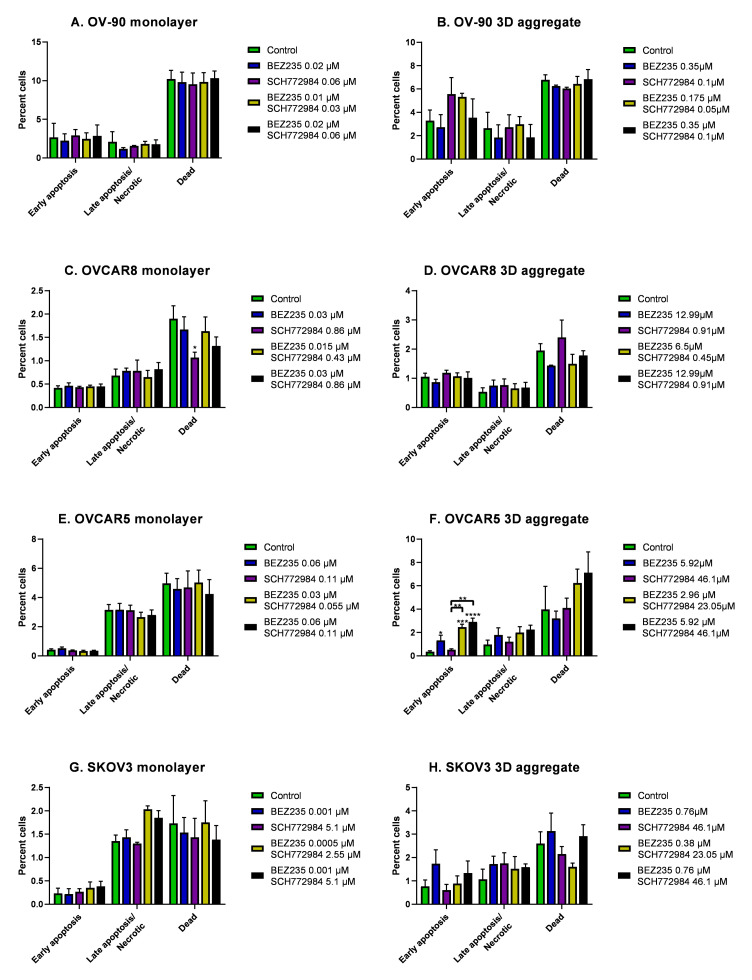
The percentage of cells in early apoptosis (Annexin+/propidium iodide (PI)-) or late apoptosis/necrotic (Annexin+/PI+) and the percentage of dead cells (Annexin-/PI+) after 24 h exposure to BEZ235 and SCH772984, alone and in combination. No significant changes were seen in the percent of early apoptosis or late apoptosis/necrotic cells and dead cells after 24 h exposure to BEZ235 and SCH772984, alone and in combination, in OV-90 monolayers or aggregates (**A**,**B**), OVCAR8 monolayers or aggregates (**C**,**D**), OVCAR5 monolayers (**E**) and SKOV3 monolayers or aggregates (**G**,**H**). The percentage of OVCAR5 aggregate cells in early apoptosis (Annexin+/PI-) increased in response to BEZ235 alone and in combination with SCH772984 (**F**). Data are expressed as mean ± SEM (n = 3). Statistical significance is at *p* < 0.05 and is in comparison to the control. The statistically significant differences are indicated as *p* < 0.05(*), *p* < 0.01 (**), *p* < 0.001 (***) and *p* < 0.0001 (****), as determined by two-way ANOVA with Tukey’s multiple comparison test on log transformed data. Any statistically significant differences between treatment groups is indicated by horizontal significance lines.

To investigate the effect of the combination of BEZ235 and SCH772984 on proliferation the four cell lines were cultured in monolayers and aggregates for 48 h then exposed for 72 h to the inhibitor combination at the concentrations and ratios as determined for each cell line and culture method and described in Section 2.5. OV-90 monolayers and aggregates showed a dose dependent decrease in proliferation in response to the combination treatment (Figure 3A,B). The monolayers were more sensitive to the combination than the aggregates. The CI values indicate a synergistic effect in both OV-90 monolayers and aggregates (Figure 3I). A significant decrease in proliferation was also seen in OVCAR8, OVCAR5 and SKOV3 monolayers and aggregates (Figure 3C–H). However, the degree of drug combination efficacy in these two cell lines was less than that in the OV-90 cell line. The reduction in proliferation in OVCAR8 and OVCAR5 cell monolayers was more pronounced than in the cell aggregates. While, CI values indicated a synergistic interaction in OVCAR8 monolayers and OVCAR5 aggregates (Figure 3I). A strongly antagonistic interaction was seen in the OVCAR8 aggregates and OVCAR5 monolayers (CI values were greater than 2 and are, therefore, not shown). CI values indicate a synergistic interaction in SKOV3 aggregates, however, as SCH772984 did not reduce proliferation in the single inhibitor treatments this effect may be the potentiation of BEZ235, rather than a synergistic effect (Figure 3I). Despite the synergistic reduction in proliferation the inhibitor combination was not found to robustly induce apoptosis, with a significant increase in apoptosis only found in OVCAR5 aggregates, where BEZ235 alone also significantly increased apoptosis (Figure 4).

### 3.4. The Effect of BEZ235 and SCH77298, Alone and in Combination, on Apoptosis in Monolayers and Aggregates of OV-90, OVCAR8, OVCAR5 and SKOV3 Cell Lines

To investigate the effect of BEZ235 and SCH772984, alone and in combination, on apoptosis, OV-90, OVCAR8, OVCAR5 and SKOV3 were established as monolayers and aggregates for 48 h before 24 h of exposure to BEZ235 and SCH772984, either alone at their respective 1× EC${}_{50}$ or at their respective 1/2× and 1× EC${}_{50}$ combinations. The percentage of cells in early apoptosis, late apoptosis/necrosis or dead was determined by Annexin-V FITC and propidium iodide staining.

Across all of the cell lines, in both the monolayer and aggregate models, the percentage of cells in apoptosis was found to be low and treatment with the inhibitors, alone and in combination, did not increase apoptosis in most of the cell lines. A statistically significant increase in apoptosis was seen in OVCAR5 aggregates, which were treated with BEZ235 alone and with the 1/2× and 1× EC${}_{50}$ combinations.

### 3.5. Morphology of OV-90 and OVCAR8 Forced Suspension Aggregates and Hanging Drop Spheroids

**Figure A1:**
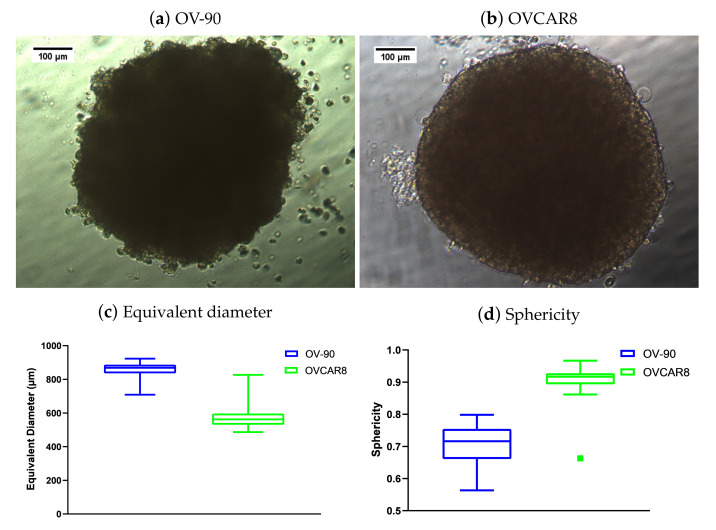
Representative images of 7-day-old spheroids produced by hanging drop culture of OV-90 (**a**) and OVCAR8 cells (**b**). Scale bar is 100 μm. A box and whisker plots showing distribution in the equivalent diameter (**c**) and sphericity (**d**) of OV-90 spheroids (blue) (n = 43) and OVCAR8 spheroids (green) (n = 35). Equivalent diameter and sphericity were determined using AnaSP.

Cell lines which are cultured in forced suspension models form cell aggregates which vary in their size and shape. OV-90 cell culture in forced suspension on polyHEMA coated plates formed small aggregates that averaged 50 ± 9.1 μm (mean ± SEM (n = 20)) in diameter. There were also a number of individual cells or clusters of just a few cells present and the majority of aggregates had an irregular shape. OVCAR8 cells formed much larger and dense aggregates, averaging 197 ± 34.2 μm (mean ± SEM (n = 19)) at their narrowest part. These aggregates had an oval shape with smoother edges and fewer single cells or smaller clusters present than in the OV-90 aggregate cultures. This variation in the size of these spheroids raised two issues. Firstly, the small loose structure of OV-90 aggregates could mean that drug penetration within the small clusters might be similar to that of the monolayers with consistent exposure to nutrients and inhibitors throughout the cell aggregates. This cell line was consistently sensitive to BEZ235 and SCH772984, alone and in combination, and showed a synergistic response to the inhibitor combination in both the 2D and 3D models. Secondly, given that size variation in this forced suspension model it is possible that the antiproliferative effect was a result of the inhibitors impacting smaller aggregates, which are likely to have higher rates of proliferation and greater exposure to inhibitors. Therefore, OV-90 and OVCAR8 cell lines were cultured using the hanging drop method to form large spheroids that had a mean equivalent diameter of 847 ± 9.0 (mean ± SEM (n = 43)) and 585 ± 13.2 μm (mean ± SEM (n = 35)), respectively, (Appendix A Figure A1c). OV-90 spheroids were less spherical than OVCAR8 spheroids with respective mean sphericity values of 0.7 and 0.9 (Appendix A Figure A1d). For the purposes of this study when OV-90 and OVCAR8 cells were grown in forced suspension they formed what is referred to as aggregates, which are small and irregularly shaped. When the cells were cultured in hanging drops they were referred to as spheroids, which were large, dense and had a more spherical morphology.

**Figure 5:**
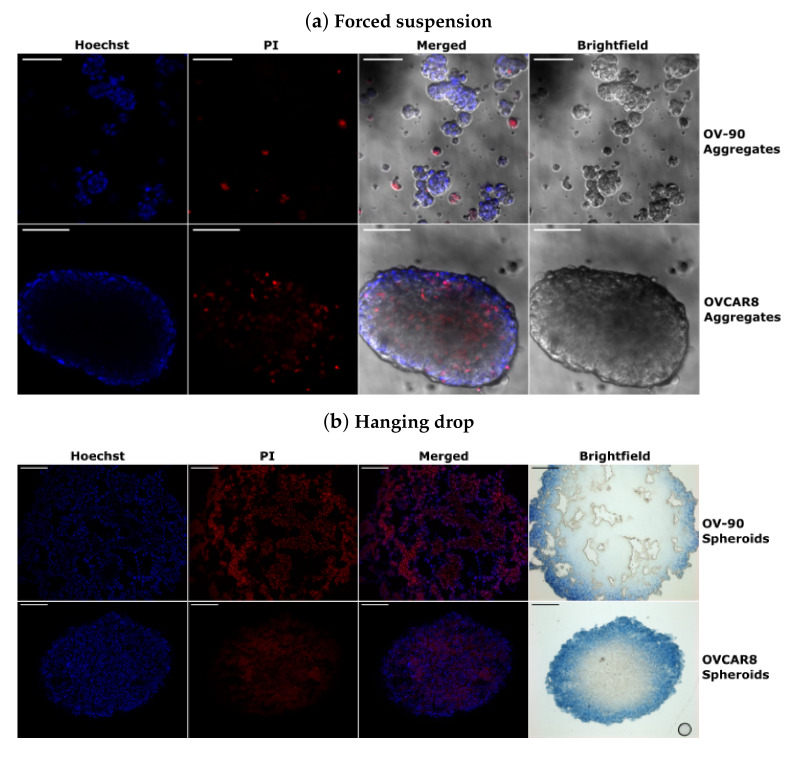
Representative fluorescent confocal microscopy images of 7-day-old aggregates produced by polyHEMA culture showing a comparison of OV-90 and OVCAR8 aggregates with propidium iodide staining (red), Hoechst 33342 staining (blue), merged and brightfield image (**a**). Representative images of 8 μm sections from the centre of 7-day-old OV-90 and OVCAR8 spheroids produced by hanging drop culture with propidium iodide staining (red), Hoechst staining (blue), merged image and brightfield image with aniline blue staining, taken using a Zeiss fluorescent microscope (**b**). Brightfield images were taken separately. Scale bar is 100 μm.

To investigate the cell density and viability of the aggregates, OV-90 and OVCAR8 cells were cultured on polyHEMA coated plates for 7 days to form aggregates. Aggregates were then stained with Hoechst and PI and imaged by fluorescent confocal microscopy. Hoechst and PI staining showed that OV-90 aggregates appeared as cell clusters often containing only a small number of cells. There were few PI positive cells and these were typically an individual cell or cells within the smaller clusters (Figure 5a). OVCAR8 aggregates were large and dense, with Hoechst staining restricted to the periphery, which is likely due to a lack of penetration of the stain in to the aggregates (Figure 5a). PI staining was greater in the interior of the OVCAR8 aggregates than the peripheral region.

To investigate cell density and viability of the spheroids OV-90 and OVCAR8 spheroids were produced by culturing in hanging drop for 7 days. Aggregates were stained with PI prior to fixing and embedding. Eight µm thick sections were taken from the center of the spheroid and stained with Hoechst and images were obtained by fluorescent microscopy. OV-90 cell formed large spheroids that contained several hollow regions and abundant PI staining (Figure 5b). In contrast, the OVCAR8 spheroids were more densely packed with cells and had less PI staining (Figure 5b). The percentage of viable cells was determined by trypan blue exclusion cell counts and showed that the majority of OV-90 cells were viable in both aggregates and spheroids (66% and 67%, respectively). The viability of OVCAR8 cells was lower in both aggregate and spheroid models (35% and 52%). This is in contrast to the low PI staining seen in OVCAR8 spheroids, which could be a result of the PI stain being less able to penetrate the dense OVCAR8 spheroids or from non-viable cells, which are not associated with the spheroids and are lost in the staining process, but not lost from the trypan blue exclusion assay.

### 3.6. Comparison of the Anti-Proliferative Effect of BEZ235 and SCH772984 in OV-90 and OVCAR8 Aggregates and Spheroids

To compare the anti-proliferative effect of BEZ235 and SCH772984 in the forced suspension aggregates and hanging drop spheroids, OV-90 and OVCAR8 aggregates and spheroids were established for 7 days on polyHEMA coated plates or in hanging drops, before treatment with the inhibitors. The establishment time in these experiments was increased to 7 days in order to allow the spheroids to form dense structures. This was also the case for the suspension aggregates in order to allow a direct comparison between the two models.

**Figure 6:**
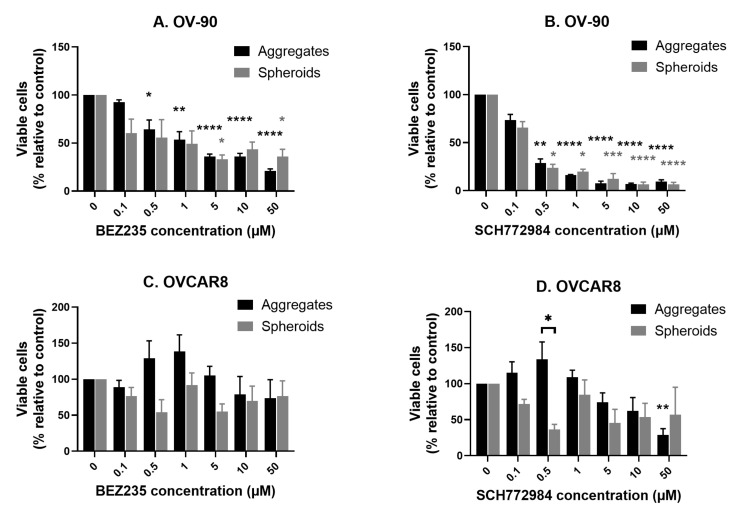
The effect of the PI3K/mTOR inhibitor BEZ235 and the ERK inhibitor SCH772984 on proliferation in OV-90 (**A**,**B**) and OVCAR8 (**C**,**D**) aggregates and spheroids. BEZ235 significantly reduced proliferation at concentrations of 0.5 μM and above in OV-90 aggregates and at 5 and 50 μM in OV-90 spheroids (**A**) but did not reduce proliferation in OVCAR8 aggregates or spheroids (**C**). SCH772984 significantly reduced proliferation at concentrations of 0.5 μM and above in OV-90 aggregates and spheroids, respectively, (**B**) and at 50 μM in OVCAR8 aggregates (**D**) but did not significantly effect proliferation in OVCAR8 spheroids. Cultures were established for 7 days prior to 72 h of exposure to the inhibitors. Data are expressed as mean ± SEM (n = 4). Statistical significance is at *p* < 0.05 and is in comparison to the control. The statistically significant differences are indicated as *p* < 0.05 (*), *p* < 0.01 (**), *p* < 0.001 (***) and *p* < 0.0001 (****), as determined by one-way ANOVA with Dunnett’s multiple comparison test on log transformed data. Any statistically significant differences between treatment groups is indicated by horizontal significance lines.

In response to BEZ235 proliferation decreased in a dose-dependent manner in OV-90 aggregates, with statistically significant reductions relative to the control at concentrations of 0.5 μM and above (Figure 6A). A similar response was seen in OV-90 spheroids although these were less sensitive with statistically significant reductions at 5 and 50 μM. There was no significant difference between the response of OV-90 aggregates and spheroids to BEZ235. At the concentrations tested BEZ235 had no significant effect on proliferation in OVCAR8 suspension aggregates or spheroids (Figure 6C) suggesting that these may be resistant to the inhibitor. A statistically significant difference was found between the response of OVCAR8 aggregates to BEZ235 in comparison to OVCAR8 spheroids ( *p* = 0.0248).

Proliferation was reduced in a dose dependent manner in OV-90 suspension aggregates and spheroids in response to SCH772984, with statistically significant reductions relative to the control at concentrations of 0.5 μM and above in both the suspension aggregates and the spheroids (Figure 6B). No significant difference was found in the response of OV-90 suspension aggregates and spheroids. A statistically significant decrease in OVCAR8 proliferation was only seen in suspension aggregates at the highest concentration tested (50 μM), suggesting that both OVCAR8 aggregates and spheroids are resistant to SCH772984 (Figure 6D). However, there was a significant difference between the response of OVCAR8 suspension aggregates and spheroids to SCH772984 (*p* = 0.0265).

The Compusyn software was used to predict EC${}_{50}$ values for the inhibitors [56]. The EC${}_{50}$ values for SCH772984 against OV-90 suspension aggregates and spheroids were comparably low. The EC${}_{50}$ value for BEZ235 against OV-90 suspension aggregates was higher than for OV-90 spheroids (2.81 vs. 0.94 μM, respectively), although, a greater reduction in proliferation was seen in the suspension aggregates (Table 2). In contrast, the predicted EC${}_{50}$ values for both inhibitors were higher in both OVCAR8 suspension aggregates and hanging drop spheroids, indicating that OVCAR8 cells in both culture conditions were resistant to BEZ235 and SCH772984. Additionally, the EC${}_{50}$ values for SCH772984 in OVCAR8 suspension aggregates increased from 0.91 to 17.46 μM with the longer establishment duration (48 h to 7 d) (Table 1 and Table 2). We were unable to test the inhibitor combinations on OVCAR8 suspension aggregates or hanging drop spheroids as the high EC${}_{50}$ values, coupled with the limited solubility of the inhibitor, would have resulted in the cells being exposed to high DMSO concentrations. For this reason the combination experiments focused on OV-90 suspension aggregates and hanging drop spheroids alone.

### 3.7. Synergistic Interaction of the Combination of BEZ235 and SCH772984 in OV-90 Aggregates and Spheroids

**Figure 7:**
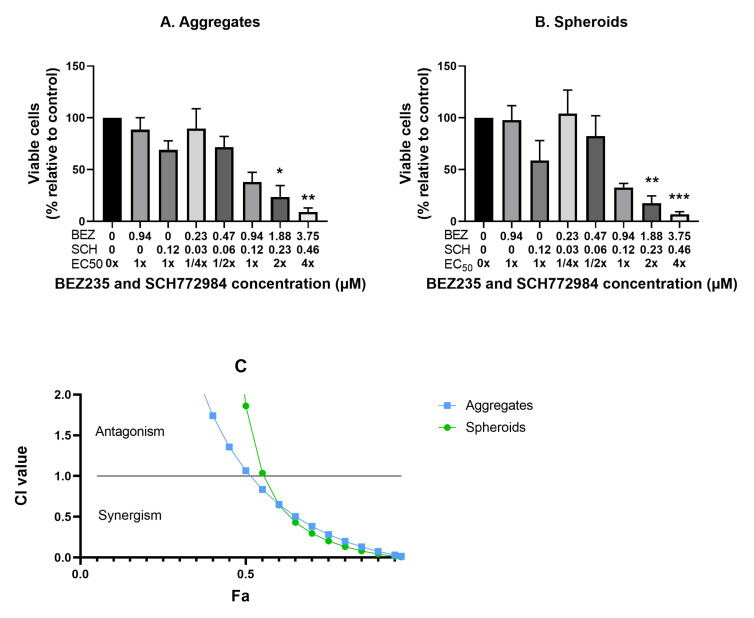
The effect of the combination of BEZ235 and SCH772984 cell proliferation in OV-90 suspension aggregates (**A**) and hanging drop spheroids (**B**). OV-90 aggregates and OV-90 spheroids were established for 7 days followed by 72 h exposure to the inhibitor combination at 1/4, 1/2, 1, 2 and 4× EC${}_{50}$. Proliferation was significantly reduced in OV-90 aggregates by the 4× EC${}_{50}$ combination and in OV-90 spheroids at the 2 and 4× EC${}_{50}$ combinations. CI values indicate a synergistic interaction in both OV-90 aggregates and spheroids (**C**). Data are expressed as mean ± SEM (n = 4). Statistical significance is at *p* < 0.05 and is in comparison to the control. The statistically significant differences are indicated as *p* < 0.05 (*), *p* < 0.01 (**) and *p* < 0.001 (***), as determined by one-way ANOVA with Tukey’s multiple comparison test on log transformed data.

To investigate the effects of the inhibitor combination on OV-90 aggregates and spheroids BEZ235 and SCH772984 were combined at the EC${}_{50}$ values derived solely from the OV-90 spheroids to enable a more direct comparison between the response of the suspension aggregates and the hanging drop spheroids (Table 2 top right column). OV-90 aggregates and spheroids were treated with BEZ235 and SCH772984 alone at their respective EC${}_{50}$ values (0.94 and 0.12 μM, respectively) and with the inhibitor combinations at constant ratios based on the EC${}_{50}$ values (Table 2). These combinations were 1/4, 1/2, 1, 2 and 4× 0.94:0.12 μM. The combination of BEZ235 and SCH772984 significantly reduced proliferation at the 4× EC${}_{50}$ combination in the OV-90 aggregates and at the 2× and 4× EC${}_{50}$ combinations in the OV-90 spheroids (Figure 7). Treatment with BEZ235 or SCH772984 alone at their EC${}_{50}$ concentrations (0.94 and 0.12 μM, respectively) did not result in a significant decrease in proliferation in OV-90 aggregates and spheroids. CI values indicate a synergistic reduction in proliferation in both OV-90 aggregates and OV-90 spheroids (Figure 7). CI values were similar between the two culture methods above F${}_{a}$ 0.6, indicating that a similar synergistic interaction was occurring under both culture conditions.

### 3.8. The Effect of BEZ235 and SCH772984, Alone and in Combination, on the Equivalent Diameter and Sphericity of OV-90 Spheroids

To investigate the effect of BEZ235 and SCH772984, alone and in combination, on the morphology of OV-90 spheroids the equivalent diameter and sphericity was tracked by imaging 7 day old spheroids prior to exposure to the inhibitors and 72 h later. AnaSP software [58] was used to analyze the changes in equivalent diameter and sphericity.

**Figure 8:**
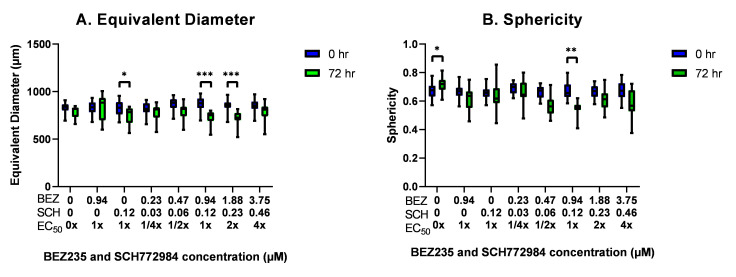
A box and whisker plot showing changes in diameter (**A**) and sphericity (**B**) after 72 h of exposure to BEZ235 and SCH772984, alone and in combination. Statistical significance is at *p* < 0.05 and is in comparison to the initial diameter or sphericity. The statistically significant differences are indicated as *p* < 0.05 (*), *p* < 0.01 (**) and *p* < 0.001 (***), as determined by RM two-way ANOVA with Sidak’s multiple comparison test (n = 3).

The equivalent diameter tended to decrease in the spheroids which were treated with SCH772984 and the inhibitor combination. This decrease was statistically significant for the spheroids treated with SCH772984 alone and the 1 and 2× EC${}_{50}$ combinations for 72 h in comparison to their initial equivalent diameter (Figure 8A). A statistically significant increase in sphericity was seen in the control group after 72h, whereas a statistically significant decrease in sphericity was seen in the 1× EC${}_{50}$ combination (Figure 8B).

## 4. Discussion

This study presents the novel combination of a PI3K/mTOR inhibitor and an ERK inhibitor as a potential treatment for ovarian cancer and directly compares the effect of these inhibitors in both monolayer and three-dimensional in vitro ovarian cancer models. The combination of BEZ235 and SCH772984 used in this present study showed a synergistic antiproliferative effect against ovarian cancer cell lines, which was cell line dependent. The ovarian cancer cell lines were also found to be less sensitive to BEZ235 and SCH772984, alone and in combination, in the 3D culture models in comparison to 2D monolayer models. Additionally, the response of the cell lines to the inhibitors was influenced by the type of 3D culture model that was used. The inhibitors had a synergistic effect reducing proliferation of OV-90 cells in 2D and in both 3D models. Conversely, the OVCAR8 cells cultured as hanging drop spheroids showed no sensitivity to SCH772984, whereas they were sensitive when grown as suspension aggregates. These findings provide a direct comparison of the anti-cancer effect of kinase inhibitors targeted at proliferation and survival signaling pathways in 2D and 3D culture models. Additionally, the potential efficacy of the novel combination of BEZ235 and SCH772984 against ovarian cancer is established, as well as SCH772984 alone, which, to our knowledge, had yet to be investigated against ovarian cancer 2D and 3D in vitro models. These findings provide support for further investigation of these inhibitors against ovarian cancers.

The combination of a PI3K/mTOR inhibitor (PF502) and MEK inhibitor (PD901) have previously been shown to have a synergistic antiproliferative effect against ovarian cancer cell lines cultured as monolayers [25]. In the present study the combination of a PI3K/mTOR inhibitor with an ERK inhibitor is shown to have a synergistic effect against both monolayer and three-dimensional ovarian cancer models. The combination of BEZ235 and SCH772984 resulted in a consistent synergistic anti-proliferative effect in OV-90 monolayers, OV-90 3D aggregates formed on polyHEMA coated plates and in OV-90 hanging drop spheroids, as determined by CI values. However, while the inhibitors had a synergistic action against OVCAR8 monolayers, a strong antagonistic interaction was seen with OVCAR8 3D aggregates. Conversely, an antagonistic interaction was seen with OVCAR5 and SKOV3 monolayers, but CI values indicated a synergistic interaction in OVCAR5 and SKOV3 3D aggregates. In SKOV3, this interaction is likely to be potentiation rather than synergism due to the lack of response to SCH772984 alone and the relative sensitivity to BEZ235. This variation in synergistic versus antagonistic effects between monolayer and aggregates models is consistent with a previous study, which tested a range of cytotoxic agents and targeted inhibitors in combination and found that the effects of drug combinations was dependent on whether a 2D or 3D colon cancer model was used [55]. It was also found that the synergistic effect of drug combinations involving a MEK inhibitor was greater in colon cancer spheroids in comparison to monolayers [55]. Determining effective combinations is a critical aspect of anticancer therapeutics where tumors eventually develop resistance to most single agent therapeutics [60]. Additionally, it is hoped that synergistic combinations could be used to overcome issues of toxicity [19,60,61]. This may be especially relevant in cases where patients are not able to tolerate higher doses due to more severe side effects. BEZ235 has shown issues with toxicity, often causing gastrointestinal side effects, so finding effective combinations with BEZ235 may help overcome these by reducing the dose that has to be administered [34,35]. Conversely, the strongly antagonistic effect seen in OVCAR8 aggregates and OVCAR5 and SKOV3 monolayers may indicate that this combination could promote tumor cell proliferation in some patients. The potential for antagonistic effects highlights the importance of patient selection and the use of biomarkers in the selection of treatment regimens. Additionally, factors such as inhibitor ratios and treatment sequence can impact drug interactions so further work is required to determine predictive biomarkers and appropriate treatment strategies [62,63,64,65].

All of the cell lines showed a reduction in proliferation in response to BEZ235 in both monolayer and aggregate models. However, a 10-fold higher concentration was required to gain a statistically significant decrease in proliferation for the cells grown in the 3D aggregate models and this effect was reflected in the higher EC${}_{50}$ values. These results are in line with previous studies which have found BEZ235 to be an effective single agent against ovarian cancer monolayers in vitro [29]. Interestingly, the ERK inhibitor SCH772984, which was developed as a treatment for melanoma resistant to BRAF targeted therapies [41], was effective at reducing proliferation in all four monolayer models and two of the four 3D aggregate models, with low EC${}_{50}$ values. This suggests that this inhibitor warrants further investigation as a potential therapeutic agent against ovarian cancer. SCH772984 had an antiproliferative effect in OVCAR5 and SKOV3 monolayer models but these cell lines became resistant in the 3D aggregate models. EC${}_{50}$ values were higher in all 3D aggregate models in comparison to EC${}_{50}$ values in monolayer models from the same cell lines. Due to the limited solubility of these inhibitors and the high EC${}_{50}$ values for some cell lines the DMSO concentration exceeded 0.1% (*v*/*v*) in the inhibitor combination experiments for SKOV3 monolayers (0.2 %) and OVCAR8 (0.56%), OVCAR5 (0.52% and SKOV3 (0.47%) aggregates. Therefore, a potential cytotoxic or cytopathic effect of DMSO cannot be ruled out in these experiments. Although, EC${}_{50}$ values were used to guide the concentrations used in the combination experiments, the range of concentrations used in this study may not capture the upper limit of the antiproliferative response for certain cell lines and may, therefore, limit the interpolation of these values.

Overall, treatment with BEZ235 and SCH772984, alone and in combination, was not found to increase apoptosis in any of the cell lines in monolayers and was only found to increase in OVCAR5 aggregates. These results suggest that targeting of the PI3K/mTOR and RAS/RAF pathways results in decreased proliferation, but does not significantly increase cell death. The effects of the inhibitors may, therefore, be predominantly cytostatic rather than cytotoxic against these cell lines. However, it is possible that higher concentrations or longer treatment times may be required to induce apoptosis. Overall rates of apoptosis in cells treated with the vehicle control were low, which is consistent with previous research [66]. Ovarian cancers cells are able to evade pro-apoptotic signals [15]. The lack of increased apoptosis in response to the inhibitors is consistent with previous studies, which have reported that BEZ235 did not induce apoptosis in ovarian cancer monolayers at concentrations of 0.1 μM, but there was an increase in apoptotic cells at concentrations above 1 μM after 96 h [67]. Others have reported no increases in apoptosis relative to control in SKOV3 monolayers at a concentration of 1 μM, despite a strong inhibitory effect on proliferation [33]. In contrast, BEZ235 has been shown to increase caspase 3 activation in OVCAR5 and SKOV3 monolayers at concentrations of 0.25 and 0.5 μM [29]. These conflicting results suggest that concentration, treatment time and assay may be important determinants of any apoptotic effect. The effect of SCH772984 on apoptosis has not been examined in ovarian cancer cell lines, however, in melanoma cell lines it increased the number of apoptotic cells, at concentrations that were approximately 3–10 times the IC${}_{50}$, although these results are not directly comparable to the present study due to the different cancer cell type [42]. The activation of an alternative cell death pathway, which was not detected by our assay may also contribute to the decrease in cell numbers seen in these aggregates. Several studies have found that ovarian cancer cell lines can undergo autophagy, instead of apoptosis, in response to targeted anti-cancer agents, which can have a protective effect for tumor cells [68,69,70,71]. However, this is speculative and requires future investigation.

The presence of activating mutations within the targeted pathways generally predict sensitivity to PI3K/AKT/mTOR and RAS/RAF/MEK/ERK pathway inhibitors [29,36]. Therefore, we would predict the LGSOC, CCOC, MOC and ENOC subtypes to show increased sensitivity to these inhibitors as they commonly have PTEN, PI3K, KRAS and BRAF mutations [72]. Interestingly, the OV-90 cell line, which is considered likely to be HGSOC in origin and that does not have mutations to either of these pathways [72], was the most sensitive to these inhibitors, both alone and in combination, with EC${}_{50}$ values at or below 0.35 μM. The OVCAR8 cell line is considered a possible HGSOC cell line and has a KRAS mutation (P121H) which may confer sensitivity to ERK inhibition [72,73]. Although the KRAS mutation P121H is rare and its significance is not well understood, it has been associated with sensitivity to MEK inhibition in OVCAR8 cells [73]. The sensitivity of the likely HGSOC cell lines, OV-90 and OVCAR8, to BEZ235 and SCH77284 alone, and the robust synergistic effect seen in OV-90 monolayers, aggregates and spheroids, suggest that the combination of these inhibitors warrants further investigation as potential ovarian cancer treatments, with 70% of all ovarian cancers of HGSOC origin [72].The OVCAR5 cell line, which has the common KRAS G12V mutation, was also found to be sensitive to both inhibitors in monolayer models but was less sensitive to BEZ235 (EC${}_{50}$ values of 0.06 vs. 5.92) and resistant to SCH772984 in the 3D aggregate models. The SKOV3 cell line, which is thought to be of CCOC origin, has a PIK3CA H1041R mutation, was sensitive to PI3K/mTOR inhibition but showed resistance to the ERK inhibitor SCH772984 [72]. This is consistent with previous research which found wild type KRAS and BRAF melanoma cells to be less sensitive to SCH772984 [41].

The forced suspension aggregate models produced cell aggregates with a large variation in both size and morphology. While this heterogeneous suspension of cell aggregates may provide a representative model of a patients ascitic fluid, which can contain aggregates of various sizes and morphology, these models do not allow for an investigation of the effect of the inhibitors on large dense aggregates. The size of 3D aggregates is an important factor in inhibitor sensitivity with increased size resulting in increased resistance [74]. Spheroids of 250 μm diameter have shown reduced drug penetration at concentrations that were 10 times higher than those used in monolayers [75]. Spheroids of 600 μm diameter show greater proliferation on the periphery, this decreases toward the interior and contains a hypoxic core. In contrast those that are 150 μm have consistent proliferation throughout and no apparent hypoxic core [76]. In the present study the OV-90 cell line was found to be sensitive to the inhibitors alone in both 2D and 3D models and had a robust synergistic response to the inhibitor combination. However, this cell line formed small loose aggregates, which often consisted of a few loosely packed cells and which were irregularly shaped. Therefore, OV-90 aggregates are unlikely to be influenced by nutrient deprivation, to form necrotic and quiescent layers or to have reduced drug penetration. In the polyHEMA forced suspension models, we cannot rule out the apparent efficacy of these inhibitors arising from the impact of these inhibitors on smaller aggregates and single cells within a treatment well. Therefore, the effects of the inhibitors was also investigated using a hanging drop model, which consistently produced large, dense spheroids and enabled the effect of the treatments on a single spheroid to be observed as outlined in Zanoni et al. [59]. This revealed that treatment with the inhibitors reduced the equivalent diameter and sphericity of the spheroids, while the spheroid remained intact, suggesting that the inhibitors may only impact the peripheral cells. Further analysis could determine if the inhibitors affect cell adhesion in the outer layer of cells as well as gradients of reduced proliferation and cell survival.

A comparison of OV-90 and OVCAR8 polyHEMA aggregates and hanging drop spheroids revealed that these cell lines formed 3D structures which varied in size and morphology. This difference was most obvious in the OV-90 cell line, which formed small loose and irregularly shaped cell clusters in the forced suspension model but will form large spheroids in hanging drop, which are consistently bigger than 700 μm in equivalent diameter. The OVCAR8 cell line formed large dense aggregates on polyHEMA plates, but these varied in size and morphology. In hanging drops OVCAR8 produced large spheroids, which were consistent in size and morphology and appeared more densely packed with cells. The structure of OV-90 and OVCAR8 spheroids in hanging drops has previously been described and were consistent with our findings [48,77]. While, OV-90 aggregates and spheroids showed similar responses to the inhibitors, both alone and in combination, the response of OVCAR8 aggregates and spheroids were significantly different. Considerable PI staining was found in the centre of OV-90 spheroids, whereas the staining in OVCAR8 spheroids appeared to be non-specific (as individual cells were not visible). However, this may be a result of the stain being less able to penetrate these spheroids due to their density. The large size of the spheroids likely resulted in a hypoxic, nutrient deprived environment towards the centre and as a result the central cells are unlikely to be alive. Increases in the size of ovarian cancer cell line spheroids have been demonstrated to reduce their sensitivity to cytotoxic agents [74,77], which is likely the result of the increased hypoxic environment, the formation of necrotic and quiescent regions, and reduced drug penetration [78]. Furthermore, previous studies have also demonstrated the ability of ovarian cancer cell lines to adapt to a hypoxic environment, resulting in changes to gene expression, evasion of apoptotic signals and increased resistance to cytotoxic agents [79,80]. The differences in response to the inhibitors between monolayer and three-dimensional models and between different three-dimensional models described in this study, coupled with these previously established characteristics suggests that not only is the use of 3D aggregate models over 2D monolayer models an important consideration in investigating the preclinical efficacy of targeted inhibitors, so is the type of 3D model that is used. While the aggregate and spheroid models used in this present study are likely to be more representative of tumor response, co-culture with stromal cells or the inclusion of ECM-mimicking hydrogels is likely to further impact the efficacy of these inhibitors through changes in gene expression [50]. This maybe particularly relevant with the use of ovarian cancer ex vivo 3D models to predict clinical response to chemotherapies being investigated [81].

## 5. Conclusions

This present study investigated the novel combination of PI3K/mTOR and ERK inhibitors against ovarian cancer and provides evidence of the potential efficacy of the combination of BEZ235 and SCH772984 against ovarian cancer with a robust synergistic interaction seen in OV-90 2D monolayers, 3D aggregates and 3D spheroids as well as OVCAR8 monolayers and OVCAR5 aggregates. Additionally, the ERK inhibitor SCH772984 shows cell line-dependent antiproliferative effects, suggesting that this inhibitor has potential as an ovarian cancer treatment. Additionally, this study provides a direct comparison of the effect of these targeted anti-proliferative inhibitors in monolayer and three-dimensional, which was yet to be investigated in ovarian cancer models. It was found that the type of model used was found to play an important role in the sensitivity of cell lines to these inhibitors with 3D aggregate models being less sensitive than their 2D monolayer counterparts. Furthermore, OVCAR8 cells grown in hanging drops differed in their response to the individual inhibitors in comparison to those grown as forced suspension aggregates. Further work is required to establish potential biomarkers to ensure these combination treatment strategies are given to patients who will benefit from it.

## Figures and Tables

**Table 1:** Table of EC${}_{50}$ (μM) values for the two inhibitors, BEZ235 and SCH772984, against OV-90, OVCAR8, OVCAR5 and SKOV3 monolayers (2D) and aggregates (3D).

	OV-90		OVCAR8		OVCAR5		SKOV3	
	**2D**	**3D**	**2D**	**3D**	**2D**	**3D**	**2D**	**3D**
BEZ235	0.02	0.35	0.03	12.99	0.06	5.92	0.001	0.76
SCH772984	0.06	0.10	0.86	0.91	0.11	46.10	5.10	1322.37

**Table 2:** Table of EC${}_{50}$ values (μM) for BEZ235 and SCH772984 against OV-90 and OVCAR8 forced suspension aggregates and hanging drop spheroids.

		Aggregates	Spheroids
OV-90	BEZ235	2.81	0.94
	SCH772984	0.14	0.12
OVCAR8	BEZ235	>50	>50
	SCH772984	17.46	>50

## Data Availability

Data available on request.

## Abbreviations

The following abbreviations are used in this manuscript:

AKT	Protein Kinase B
BRAF	Serine/threonine-protein kinase B-Raf
BSA	Bovine serum albumin
CCOC	Clear cell ovarian carcinoma
CI	Combination index
DMEM-F12	Dulbecco’s Modified Eagle Medium/Nutrient Mixture F-12
DMSO	Dimethyl sulfoxide
ECM	Extra cellular matrix
EDTA	Ethylenediaminetetraacetic acid
ENOC	Endometrioid ovarian carcinoma
EOC	Epithelial ovarian carcinoma
ERBB	Epidermal growth factor receptor
ERK	Extracellular signal-related kinase
FBS	Foetal bovine serum
HGSOC	High grade serous ovarian carcinoma
KRAS	Kirsten rat sarcoma virus
LGSOC	Low grade serous ovarian carcinoma
MAPK	Mitogen-activated protein kinase
MDPI	Multidisciplinary Digital Publishing Institute
MEK	Mitogen-extracellular activated protein kinase kinase
mTOR	Mammalian or mechanistic target of rapamycin inhibitor
MOC	Mucinous ovarian carcinoma
p-AKT	Phosphorylated Protein Kinase B
PARP	Poly(ADP-Ribose) Polymerase
PBS	Phosphate buffered saline
p-ERK	Phosphorylated Extracellular signal-related kinase
PI	Propidium iodide
PI3K	Phosphoinositide 3-kinase
PIK3CA	Phosphatidylinositol-3-kinase 110-kDa catalytic subunit
poly-HEMA	Poly-hydroxyethylmethacrylate
PTEN	phosphatase and tensin homologue deleted on chromosome ten
PVDF	Polyvinyl difluoride
RAF	V-raf murine sarcoma viral oncogene homolog
RAS	Rat sarcoma viral oncogene homolog
RIPA	Radio immunoprecipitation assay
SDS	Sodium dodecyl sulphate
SDS-PAGE	Sodium dodecyl sulphate poly-acrylamide gel electrophoresis
TBS	Tris-buffered saline
TBS-t	Tris-buffered saline and tween 20
